# Phase 1 randomized trial of HS-10353, a novel GABA(A) positive allosteric modulator for treatment of major depressive disorder

**DOI:** 10.1186/s12916-026-04926-5

**Published:** 2026-05-21

**Authors:** Zhenlei Wang, Mingli Li, Xiaojin Xu, Ruiling Zhang, Huali Lin, Fude Yang, Jie Qi, Huifeng Zhang, Yuying Tao, Qi Shen

**Affiliations:** 1 Mental Health Center, West China Hospital, Sichuan University, Chengdu, China; 2 Clinical Trial Center, West China Hospital, Sichuan University, Chengdu, China; 3 Department of Psychiatry, Wuhan Mental Health Center, Wuhan, China; 4 Department of Psychiatry, Henan Mental Hospital, Xinxiang, China; 5 Xi’an Mental Health Center, Xi’an, China; 6 Beijing HuiLongGuan Hospital, Capital Medical University, Peking University HuiLongGuan Clinical Medical School, Beijing, China; 7 Shanghai Hansoh BioMedical Co., Ltd., Shanghai, China

**Keywords:** HS-10353, Major Depressive Disorder, Tolerability, Efficacy, Pharmacokinetics

## Abstract

**Background:**

HS-10353 is a novel positive allosteric modulator of γ-aminobutyric acid A receptors that is under development for the treatment of major depressive disorder (MDD).

**Methods:**

This is a double-blind, randomized, placebo-controlled, phase 1 trial comprising single ascending dose (SAD) and multiple ascending dose (MAD) parts. In SAD, 8 healthy volunteers of each cohort were randomized (6:2) to receive HS-10353 (2–55 mg) or placebo in a fasted state. In MAD, 12 MDD patients of each cohort were randomized (9:3) to receive HS-10353 (15–65 mg/day given for up to 7 days) or placebo. The primary objective was to assess the safety and tolerability of HS-10353. The efficacy endpoint in the MAD part included the change in the total score of the 17-item Hamilton Rating Scale for Depression (HAM-D17) at day 8. Pharmacokinetic (PK) analysis was performed as a secondary objective.

**Results:**

Forty-eight healthy participants and forty-eight MDD participants completed the study. The most frequently observed treatment emergent adverse events (TEAEs) in the HS-10353 dose groups (occurring in ≥ 3 cases) included white blood cells urine positive and alanine aminotransferase increased in the SAD part, white blood cell count decreased and somnolence in the MAD part. All adverse events (AEs) were mild to moderate, and there were no serious adverse events or AEs that led to withdrawal from the trial. Treatment with 50 mg of HS-10353 for only 7 days showed therapeutic effects, with a difference of -4.7 (95% CI: -8.9 to -0.5) in change from baseline of HAM-D17 score on day 8 compared to placebo. HS-10353 exhibited a terminal half-life of 14 to 22 h with minimal accumulation upon repeat dosing (approximately 1.3- to 2.5-fold), supporting once-daily dosing; while absorption saturation was observed at fasting dose levels greater than 45 mg.

**Conclusions:**

Single and multiple oral doses of HS-10353 were safe and well tolerated with a favorable PK profile. The findings in this small, short duration study suggest an early signal in patients with MDD that should be further investigated.

**Trial registration:**

The trial is registered at China Drug Trials (CTR20210105, 2021-01-21) and ClinicalTrials.gov (NCT05195203, 2022-01-13).

## Background

Major depressive disorder (MDD) is a leading cause of ill health and disability, and over 264 million people worldwide are affected by MDD in 2018 [1, 2]. The World Health Organization (WHO) has ranked MDD as the third leading cause of global disease burden, and projects that MDD will become the primary cause by 2030 [1]. MDD is widely recognized as a multifactorial condition, encompassing biological, genetic, psychosocial, and environmental factors. It is characterized by persistent low or depressed mood and/or diminished interest in previously enjoyable activities, accompanied by additional symptoms such as significant weight loss and insomnia [1, 3]. The first-line pharmacological treatment for MDD, including monoamine oxidase inhibitors (MAOIs), selective serotonin reuptake inhibitors (SSRIs), and serotonin–norepinephrine reuptake inhibitors (SNRIs), have been available for a significant duration of time. However, these treatments result in remission in only 40% of MDD patients, with the onset of remission typically delayed by several weeks to several months [1, 3, 4]. Additionally, at least 50% of patients develop resistance to treatment, accompanied by a range of side effects that compromise overall well-being and adherence to therapy [4]. Esketamine was recently approved by US Food and Drug Administration (FDA) for the treatment of treatment-resistant depression (TRD) in adults, as monotherapy or in conjunction with an oral antidepressant. The efficacy was initially observed at 24 h post–first dose and were maintained through day 28 of the double-blind treatment phase [5]. But esketamine has the risk of physical and psychological dependence and is only available through a restricted program [6]. Therefore, it is urgent need for new antidepressants with rapid onset of action and less side effects.

In recent years, an enhanced understanding of the etiology of MDD and its treatments has prompted the exploration of synthetic neuroactive steroids and their analogs as potential therapeutic agents for postpartum depression (PPD). These compounds modulate γ-aminobutyric acid (GABA) receptors, which constitute the primary inhibitory signaling system in the central nervous system (CNS) [1, 3, 4, 7]. It has been reported that patients with MDD exhibit significantly lower levels of GABA in the cerebrospinal fluid, occipital cortex, prefrontal cortex, and plasma [8–10]. Accumulating evidence supports the hypothesis that depression may be associated with a deficiency in GABA and subsequent alterations in downstream monoaminergic neurotransmission [11]. This suggests that GABA, or positive allosteric modulators of GABA receptors, which are the primary receptors for this inhibitory neurotransmitter, could represent a promising target for the development of novel antidepressants [11]. Additionally, allopregnanolone, a naturally occurring neurosteroid and endogenous positive allosteric modulator of both synaptic and extrasynaptic GABA_A_ receptors, enhances neuronal inhibition by prolonging the opening time of GABA-activated chloride channels [12, 13]. Therefore, one strategy for the development of antidepressant drugs is to enhance the levels of allopregnanolone in patients. In recent years, brexanolone injection, a soluble mixture of allopregnanolone and sulfobutylether-β-cyclodextrin, was approved by FDA for the treatment of PPD in adults in 2019 [14].

However, the application of brexanolone is limited by its requirement for intravenous administration [14, 15]. Notably, zuranolone (SAGE-217), an orally administered synthetic neurosteroid and positive allosteric modulator of GABA_A_ receptors, was approved by FDA for PPD and by Japan’s PMDA for both PPD and MDD [2, 16, 17]. Zuranolone demonstrates a more rapid onset of action compared to conventional antidepressants used for PPD, with a dosing regimen of once daily for only two weeks. This profile presents a significant advancement and provides substantial motivation for pharmaceutical companies to develop more effective treatments for PPD [2, 16, 18–21].

Similar to the mechanism of zuranolone, HS-10353 is a novel Class 1 chemical drug that has been independently developed by Hansoh Pharmaceutical Group Co., Ltd. (Shanghai, China). HS-10353 exhibits potent activation effects on GABAA1 (α1β2γ2) ion channel currents, comparable to that of zuranolone. Additionally, HS-10353 demonstrates significantly stronger activation of GABAA4 (α4β3δ) ion channel currents, with a potency marginally higher than that of zuranolone. Furthermore, HS-10353 demonstrated good tolerability in animal toxicology studies (rats and dogs), indicating favorable safety profiles. Based on preclinical favorable efficacy and safety profiles, HS-10353 received approval from China’s National Medical Products Administration (NMPA) for clinical investigation in 2021. Currently, HS-10353 is undergoing Phase 2 clinical trials (NCT05937867). This report details the safety and efficacy findings of its Phase 1 clinical trial conducted in healthy volunteers and MDD patients.

## Methods

The Phase 1 trial of HS-10353 comprised a single ascending dose (SAD) part in healthy volunteers and a multiple ascending dose (MAD) part in patients with MDD (ClinicalTrials.gov identifier: NCT05195203). This trial was a multicenter clinical trial and mainly conducted at the Clinical Trial Center of West China Hospital, Sichuan University (Chengdu, China), as the lead institution, adhering to the International Conference on Harmonization Good Clinical Practice guidelines and the principles outlined in the Declaration of Helsinki. The trial protocol was reviewed and approved by the Institutional Ethics Committee of West China Hospital, Sichuan University (Chengdu, China). All participants provided written informed consent prior to screening and enrollment.

### Study procedures and conduct

In the SAD part, healthy volunteers aged 18 to 45 years were eligible for participation if they could adequately comprehend the study content, procedures, and potential adverse reactions, and voluntarily sign the informed consent form. Eligibility criteria also included a body weight of ≥ 50 kg for males or ≥ 45 kg for females, with a Body Mass Index (BMI) within the range of 18 to 26 kg/m². Exclusion criteria comprised any potentially clinically significant disease, a history of drug allergy, use of any medication within two weeks prior to or during the trial, clinically significant abnormalities in electrocardiogram (ECG), or an absolute QTc interval corrected by Fridericia’s formula (QTcF) exceeding 450 milliseconds for males and 470 milliseconds for females. Based on the no observed adverse effect level (NOAEL) values and the maximum tolerated doses (MTD) from 4-week toxicology studies in rats and dogs, the SAD part commenced with an initial dose of 2 mg, escalating sequentially in 5 dose cohorts (2, 6, 15, 30, 45, and 55 mg). Within each dose cohort, participants were randomized in a 3:1 ratio to receive either a single oral dose of HS-10353 capsule (*n* = 6) or placebo (*n* = 2) under fasting conditions ( ≥ 10 h of overnight fasting before dosing, no food for 4 h after dosing, and water restriction from 1 h pre-dose to 1 h post-dose). For safety considerations, a sentinel dosing strategy was implemented in first 2 mg cohort. Furthermore, the 30 mg cohort incorporated a chronopharmacology component to explore the PK characteristics of different dosing times (daytime vs. nighttime). Participants received their initial dose at 8:00 a.m. on Day 1, followed by a 7-day washout period, and subsequently received a second dose at 8:00 p.m. on Day 8.

In the MAD part, participants were adults aged 18 to 65 years diagnosed with MDD according to the Diagnostic and Statistical Manual of Mental Disorders, Fifth Edition (DSM-5) [22]. Eligible patients included those with recurrent episodes of MDD without psychotic features or a single episode that had persisted for at least four weeks who were treatment naïve or willing to discontinue other antidepressants, anxiolytics, or antipsychotics during the screening and treatment period. Diagnosis was confirmed through clinical evaluation and the Mini International Neuropsychiatric Interview (M.I.N.I. 7.0.2) [23]. Additionally, patients were required to have a score of 22 or higher on the 17-item Hamilton Depression Rating Scale (HAM-D17) prior to dosing. Key exclusion criteria for the MAD part comprised: homicidal ideation or intent, or suicidal ideation with a plan to act within 6 months prior to screening; clinically significant medical conditions including neuropsychiatric, cardiovascular, urinary, digestive, respiratory, musculoskeletal, metabolic endocrine, hematologic, immunologic, dermatologic, and neoplastic diseases; a history of treatment-resistant depression (TRD); a history of drug allergy or hypersensitivity to components of the investigational drug or other antipsychotic agents; and ECG abnormalities, specifically an absolute QTcF interval exceeding 450 milliseconds in males and 470 milliseconds in females, the use of any psychotropic medications within 14 days prior to screening. Based on the safety results from the SAD phase, participants in the MAD part were sequentially allocated into one of four dosing cohorts: 15 mg, 30 mg, 50 mg, and 65 mg. Within each dose cohort, participants were randomized in a 3:1 ratio to receive either HS-10353 capsule (*n* = 9) or placebo (*n* = 3) once daily under fasting conditions for 7 consecutive days as monotherapy with concomitant medications strictly prohibited except for necessary therapies to manage emergent adverse events during the trial. If the pre-specified stopping criteria are triggered during dose escalation, the dose escalation will be terminated, and the previous dose will be determined whether to be the MTD for this part, or the safety review committee will decide whether to choose a lower dose or continue with the current dose based on the specific adverse events.

### Endpoints

The primary objective of the SAD and MAD parts was to assess the safety and tolerability of HS-10353 in healthy adult Chinese volunteers and MDD patients. Secondary objectives comprised the pharmacokinetics (PK) of HS-10353 in both populations and the preliminary efficacy in MDD patients (MAD part).

Key safety endpoints included treatment-emergent adverse events (AEs), treatment-emergent serious adverse events (SAEs), clinical laboratory assessments (comprising hematology, urinalysis, blood biochemistry, coagulation function, and thyroid function), vital signs, physical examinations, ECG, Stanford Sleepiness Scale (SSS), Modified Observer’s Assessment of Alertness/Sedation (MOAA/S), and Columbia-Suicide Severity Rating Scale (C-SSRS). Clinical laboratory assessments were performed at screening, baseline (Day -1), 48 h post-dose, and on the discharge day (Day 6) in the SAD part, and at screening, baseline (Day -1), and on Days 3, 7, and 12 in the MAD part, with AEs monitored continuously throughout the study. The clinical significance of lab tests was determined by the investigators.

In the MAD, the efficacy evaluation included several endpoints: change in HAM-D17 score from baseline, HAM-D17 response rate, HAM-D17 remission rate, change in Clinical Global Impression-Severity (CGI-S) score from baseline, and change in Hamilton Rating Scale for Anxiety (HAM-A) score from baseline. Efficacy assessments were performed at pre-dose as the baseline and three time points post-dose: day 3, day 8, and day 12. The HAM-D17, CGI-S, and HAM-A scores at post-dose time points were compared to baseline values. Furthermore, the response and remission rates of the HAM-D17 score were calculated for both the study drug group and the placebo group, and between-group differences in these rates were also analyzed. The study defined HAM-D response as having a 50% or greater reduction from baseline in HAM-D total score and HAM-D remission defined as having a HAM-D total score of ≤ 7.

In the SAD part, blood samples were collected at pre-dose and 0.25, 0.5, 1, 1.5, 2, 2.5, 3, 3.5, 4, 6, 8, 10, 12, 24, 48, 72, 96 and 120 h post-dose. In the MAD part, blood samples were collected at pre-dose and predefined time points up to 24 h on day 1; and days 4, 5 and 6 (at trough); at pre-dose and predefined time points up to 120 h on day 7. Pre-dose assessments were performed within 1 h prior to dosing. Blood samples for pharmacokinetic analysis were collected via an indwelling catheter (4 mL of whole blood per time point) and were promptly centrifuged at 10,400 g for 10 min at 4°C to obtain plasma, which was subsequently stored at −80°C before analysis. The plasma concentration of HS-10353 was quantified using a validated liquid chromatography-tandem mass spectrometry (LC-MS/MS) method. In brief, the validated method was linearity over the concentration range of 1.00 to 1000 ng/mL using a plasma sample volume of 30 μL.

### Randomization and blinding

In the SAD part, six dose groups are predefined, with 8 subjects in each group. Each dose group uses a block randomization method to randomly assign subjects to the trial group or the control group at a 3:1 ratio (6 subjects in the HS-10353 group, 2 subjects in the placebo group). The first dose group (2 mg) will use gender stratification, with male and female subjects randomly assigned to the HS-10353 group (3 males and 3 females) and the placebo group (1 male and 1 female) at a 1:1 ratio.

In the MAD part, four dose groups were predefined, each with 12 subjects. Block randomization was used to randomly assign subjects to the treatment group or placebo group at a 3:1 ratio (9 subjects in the HS-10353 group and 3 in the placebo group).

This trial uses a double-blind design, under which both the subjects and the site investigators will be unaware of the subjects’ randomization results throughout the study period.

### Statistical analysis

Descriptive statistics were employed for the analysis of safety and efficacy. Unless otherwise specified, all statistical analyses were conducted using SAS version 9.4. The sample size for this trial was determined empirically, as both safety and efficacy analyses were considered exploratory in nature. Safety endpoints in the SAD and MAD studies were summarized separately by dose cohort: pooled placebo cohort, individual HS-10353 dose cohorts, and pooled HS-10353 cohorts. Efficacy endpoints in the MAD part will be summarized according to dose group. PK parameters of HS-10353 were calculated by non-compartmental analysis (NCA) with Phoenix WinNonlin (version 8.3; Certara, Princeton, NJ, USA). The PK parameters for a single dose of HS-10353 included maximum observed plasma concentration (C_max_), time to reach C_max_ (T_max_), area under the plasma concentration-time curves from the time of dosing to the time of the last observation (AUC_0-t_) and to infinity (AUC_0-∞_), elimination rate constant (λ_z_), terminal phase elimination half-life (t_1/2_), apparent clearance (CL/F), apparent volume of distribution (V_d_/F), mean residence time (MRT). Similarly, the PK parameters of the MAD part included steady-state maximum concentration (C_ss, max_), steady-state minimum concentration (C_ss, min_), steady-state average concentration (C_ss, av_), time to maximum concentration at steady state (T_ss, max_), area under the steady-state plasma concentration-time curve (AUC_ss, 0-τ_), accumulation ratio of multiple doses (R_ac_).

## Results

### Baseline characteristics of study participants

**Fig. 1:**
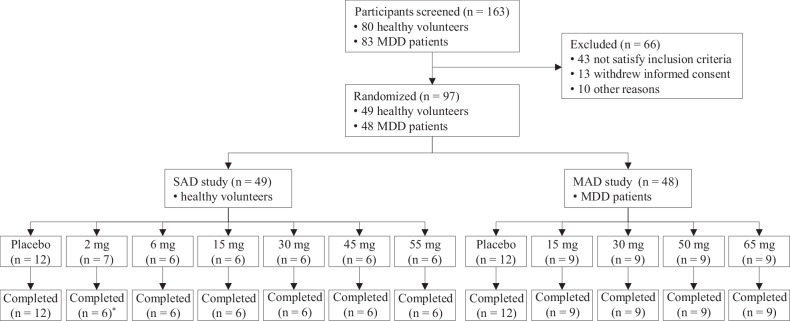
Participant screening and randomization flowchart. ^*^One Participant withdrew informed consent after randomization (before drug administration)

Between January 27, 2021, and March 31, 2023, a total of 80 healthy volunteers and 83 patients diagnosed with depressive disorder were screened for inclusion in the SAD part and MAD part, respectively (Fig. 1). A total of 66 individuals experienced screen failure across both parts. The reasons for exclusion or screen failure were as follows: 43 cases (65.2%) did not satisfy the inclusion or exclusion criteria; 13 cases (19.7%) withdrew informed consent; and 10 cases (15.2%) were excluded due to other reasons. Additionally, one participant withdrew informed consent after randomization but before administration in the SAD part. Ultimately, 48 healthy participants and 48 MDD participants completed the SAD and MAD parts, respectively. Table 1 and Table 2 present the baseline characteristics of healthy volunteers and MDD patients. In summary, the participants in the SAD part exhibited a mean age of 26.7 years (SD = 4.5), an average body weight of 61.3 kg (SD = 9.1), and a mean body mass index (BMI) of 22.4 kg/m² (SD = 2.1). In the MAD part demonstrated a mean age of 33.1 years (SD = 12.2), an average body weight of 61.3 kg (SD = 12.1), and a mean BMI of 22.3 kg/m² (SD = 3.3).

**Table 1:** The demographics and baseline characteristics of participants in the SAD part (full analysis set)

Characteristics	2 mg (*N* = 6)	6 mg (*N* = 6)	15 mg (*N* = 6)	30 mg (*N* = 6)	45 mg (*N* = 6)	55 mg (*N* = 6)	Total HS-10353 (*N* = 36)	Placebo (*N* = 12)
Age, year								
Mean (SD)	27.0 (5.4)	29.2 (5.7)	24.3 (3.9)	25.0 (1.1)	27.8 (6.7)	27.5 (2.9)	26.8 (4.7)	26.3 (4.0)
Median (range)	26.0 (22–37)	31.0 (20–36)	24.0 (20–31)	25.0 (24–26)	26.0 (23–41)	27.5 (23–31)	26.0 (20-41)	25.5 (20–36)
Ethnicity, Asian, n (%)	6 (100)	6 (100)	6 (100)	6 (100)	6 (100)	6 (100)	36 (100)	12 (100)
Sex, n (%)								
Male	3 (50.0)	4 (66.7)	5 (83.3)	3 (50.0)	4 (66.7)	4 (66.7)	23(63.9)	6 (50.0)
Female	3 (50.0)	2 (33.3)	1 (16.7)	3 (50.0)	2 (33.3)	2 (33.3)	13(36.1)	6 (50.0)
Weight, kg, mean (SD)	56.4 (8.1)	64.3 (7.4)	67.1 (10.5)	63.6 (8.6)	57.8 (8.2)	63.4 (6.4)	62.1 (8.6)	58.7 (10.3)
BMI, kg/m^2^, mean (SD)	21.7 (2.6)	23.7 (1.0)	23.1 (2.2)	22.5 (1.9)	21.4 (2.0)	22.1 (2.1)	22.4 (2.0)	22.4 (2.5)

Each dose group uses a block randomization method to randomly assign subjects to the HS-10353 or placebo at a 3:1 ratio. The first dose group (2 mg) used gender stratification, with male and female subjects randomly assigned to the HS-10353 group (3 males and 3 females) and the placebo group (1 male and 1 female) at a 1:1 ratio

*SD*, standard deviation, *BMI*, body mass index

**Table 2:** The demographics and baseline characteristics of participants in the MAD part (full analysis set)

Characteristics	15 mg QD (*N* = 9)	30 mg QD (*N* = 9)	50 mg QD (*N* = 9)	65 mg QD (*N* = 9)	Total HS-10353 (*N* = 36)	Placebo QD (*N* = 12)
Age, year						
Mean (SD)	29.6 (8.9)	37.7 (14.0)	35.3 (13.9)	37.6 (13.5)	35.0 (12.6)	27.4 (8.8)
Median (range)	28.0 (20–49)	35.0 (21–64)	28.0 (21–55)	30.0 (24–57)	30.0 (20-64)	25.0 (18–45)
Ethnicity, Asian, n (%)	9 (100)	9 (100)	9 (100)	9 (100)	36 (100)	12 (100)
Sex, n (%)						
Male	4 (44.4)	5 (55.6)	2 (22.2)	6 (66.7)	17(47.2)	6 (50.0)
Female	5 (55.6)	4 (44.4)	7 (77.8)	3 (33.3)	19(52.8)	6 (50.0)
Weight, kg, mean (SD)	62.0 (14.4)	60.4 (13.2)	55.6 (6.6)	66.0 (5.4)	61.0 (10.9)	62.24 (15.9)
BMI, kg/m^2^, mean (SD)	22.8 (3.7)	22.1 (3.3)	21.1 (2.3)	23.4 (2.7)	22.4 (3.0)	22.2 (4.2)
Baseline HAM-D17 score, mean (SD)	25.9 (2.6)	23.7 (1.4)	24.7 (2.2)	24.7 (2.5)	24.7 (2.28)	24.7 (2.6)
Baseline HAM-A score, mean (SD)	22.8 (8.1)	17.3 (5.1)	25.8 (7.6)	20.3 (7.8)	21.6 (7.61)	19.8 (7.2)
Baseline CGI-S score, mean (SD)	4.7 (0.7)	4.8 (0.7)	4.3 (0.7)	4.3 (0.5)	4.5 (0.65)	4.2 (0.6)

Each dose group uses a block randomization method to randomly assign subjects to the HS-10353 or placebo at a 3:1 ratio

*SD,* standard deviation, *BMI,* body mass index, *HAM-D17,* hamilton depression rating scale score, *HAM-A,* hamilton anxiety scale score, *CGI-S,* clinical global impression-severity score

### Safety and tolerability

A summary of AEs that occurred in participants of the SAD (daytime dosing only) and MAD parts is provided in Table 3 and Table 4 respectively. In summary, single doses of HS-10353 up to 55 mg administered to healthy volunteers and 7 daily doses up to 65 mg administered to patients with depressive disorder were well tolerated, with no SAEs or AEs leading to dose adjustment observed.

**Table 3:** Events of adverse events occurring in the SAD part (safety analysis set)

Adverse event, n (%)	HS-10353							Placebo
	2 mg (*N* = 6)	6 mg (*N* = 6)	15 mg (*N* = 6)	30 mg (*N* = 6)	45 mg (*N* = 6)	55 mg (*N* = 6)	Total (*N* = 36)	(*N* = 12)
Any TEAEs	1 (16.7)	1 (16.7)	3 (50.0)	1 (16.7)	3 (50.0)	1 (16.7)	10 (27.8)	2 (16.7)
White blood cells urine positive	0	0	1 (16.7)	1 (16.7)	2 (33.3)	0	4 (11.1)	0
Alanine aminotransferase increased	0	0	1 (16.7)	0	1 (16.7)	1 (16.7)	3 (8.3)	1 (8.3)
Protein urine present	1 (16.7)	0	1 (16.7)	0	0	0	2 (5.6)	0
Aspartate aminotransferase increased	0	0	1 (16.7)	0	1 (16.7)	0	2 (5.6)	0
Pruritus	0	1 (16.7)	1 (16.7)	0	0	0	2 (5.6)	0
Gamma-glutamyltransferase increased	0	0	0	0	1 (16.7)	0	1 (2.8)	0
Red blood cells urine positive	1 (16.7)	0	0	0	0	0	1 (2.8)	0
Blood alkaline phosphatase increased	0	0	0	0	1 (16.7)	0	1 (2.8)	0
Blood fibrinogen increased	0	0	0	0	1 (16.7)	0	1 (2.8)	0
Rash	0	1 (16.7)	0	0	0	0	1 (2.8)	0
Thyroiditis subacute	0	0	0	0	1 (16.7)	0	1 (2.8)	0
Blood glucose increased	0	0	0	0	0	0	0	1 (8.3)

*TEAE,* treatment emergent adverse event

**Table 4:** Events of adverse events occurring in the MAD part (safety analysis set)

Adverse event, n (%)	HS-10353					Placebo QD
	15 mg QD (*N* = 9)	30 mg QD (*N* = 9)	50 mg QD (*N* = 9)	65 mg QD (*N* = 9)	Total (*N* = 36)	(*N* = 12)
Any TEAEs	5 (55.6)	6 (66.7)	6 (66.7)	3 (33.3)	20 (55.6)	5 (41.7)
White blood cell count decreased	0	1 (11.1)	2 (22.2)	0	3 (8.3)	0
Somnolence	0	0	3 (33.3)	0	3 (8.3)	2 (16.7)
Alanine aminotransferase increased	1 (11.1)	0	0	1 (11.1)	2 (5.6)	0
Blood creatine phosphokinase increased	1 (11.1)	1 (11.1)	0	0	2 (5.6)	0
Neutrophil count decreased	0	1 (11.1)	1 (11.1)	0	2 (5.6)	0
Urinary tract infection	0	1 (11.1)	1 (11.1)	0	2 (5.6)	0
Upper respiratory tract infection	0	0	0	2 (22.2)	2 (5.6)	0
Constipation	0	2 (22.2)	0	0	2 (5.6)	0
Sinus bradycardia	2 (22.2)	0	0	0	2 (5.6)	1 (8.3)
Vertigo	0	2 (22.2)	0	0	2 (5.6)	0
Red blood cell count decreased	0	1 (11.1)	0	0	1 (2.8)	0
Lymphocyte count decreased	0	0	0	1 (11.1)	1 (2.8)	0
White blood cells urine positive	1 (11.1)	0	0	0	1 (2.8)	0
Blood pressure systolic decreased	0	0	1 (11.1)	0	1 (2.8)	0
Blood pressure diastolic decreased	0	0	1 (11.1)	0	1 (2.8)	0
Blood pressure diastolic increased	0	0	1 (11.1)	0	1 (2.8)	0
Blood triglycerides increased	0	1 (11.1)	0	0	1 (2.8)	0
Haemoglobin decreased	0	1 (11.1)	0	0	1 (2.8)	0
Oxygen saturation decreased	0	0	0	1 (11.1)	1 (2.8)	0
Headache	0	0	1 (11.1)	0	1 (2.8)	1 (8.3)
Dizziness	0	1 (11.1)	0	0	1 (2.8)	0
Gastroenteritis	0	1 (11.1)	0	0	1 (2.8)	0
Dry mouth	0	1 (11.1)	0	0	1 (2.8)	1 (8.3)
Nausea	0	1 (11.1)	0	0	1 (2.8)	0
Retching	0	1 (11.1)	0	0	1 (2.8)	0
Vomiting	0	1 (11.1)	0	0	1 (2.8)	0
Hypertriglyceridemia	0	1 (11.1)	0	0	1 (2.8)	0
Hepatic failure	0	0	1 (11.1)	0	1 (2.8)	0
Arthralgia	0	0	1 (11.1)	0	1 (2.8)	0
Oedema peripheral	0	1 (11.1)	0	0	1 (2.8)	0
Proteinuria	0	0	1 (11.1)	0	1 (2.8)	0
Anaemia	0	1 (11.1)	0	0	1 (2.8)	0
Dry eye	0	1 (11.1)	0	0	1 (2.8)	0
Head discomfort	0	0	0	0	0	1 (8.3)
Diarrhea	0	0	0	0	0	1 (8.3)

*TEAE,* treatment emergent adverse event

In the SAD part with daytime dosing, 10 out of 36 (27.8%) HS-10353-treated participants reported one or more AEs, while 2 out of 12 (16.7%) placebo-treated participants experienced one or more AEs (risk difference: 11.1%; 95% CI: -19.4 to 31.3). All AEs were categorized as mild in severity. Notably, in the 30 mg cohort of nighttime dosing, none of the six HS-10353-treated participants reported any AEs, whereas one AE (somnolence) was reported in two placebo-treated participants. The most frequently reported treatment emergent adverse events (TEAEs) among participants treated with HS-10353 versus placebo were white blood cells urine positive (11.1% vs 0), alanine aminotransferase increased (8.3% vs 8.3%), protein urine present (5.6% vs 0), aspartate aminotransferase increased (5.6% vs 0) and pruritus (5.6% vs 0). Notably, alanine aminotransferase increased (8.3%) was also the most common TEAE in the placebo group. All abnormal values for liver enzymes (ALT, AST, GGT, ALP) and fibrinogen are tabulated in Additional File 1: Table S1.

In the MAD part, among the 36 participants who received HS-10353, 20 (55.6%) reported at least one AE, while among the 12 participants who received placebo, 5 (41.7%) reported at least one AE (risk difference: 13.9%; 95% CI: -17.0 to 40.7). All AEs were categorized as mild to moderate in severity. No serious or life-threatening adverse events were observed during the study period. The most frequently reported TEAEs with HS-10353 versus placebo included white blood cell count decreased (8.3% vs 0), somnolence (8.3% vs 16.7%), alanine aminotransferase increased (5.6% vs 0), blood creatine phosphokinase increased (5.6% vs 0), neutrophil count decreased (5.6% vs 0), urinary tract infection (5.6% vs 0), upper respiratory tract infection (5.6% vs 0), constipation (5.6% vs 0), sinus bradycardia (5.6% vs 8.3%) and vertigo (5.6% vs 0). In contrast, the most common TEAEs in the placebo group were somnolence (16.7%). Notably, the incidence of TEAEs was not dose-dependent (15 mg: 55.6%; 30 mg: 66.7%; 50 mg: 66.7%; 65 mg: 33.3%), and no significant differences were observed in AE rates across the HS-10353-treated cohorts compared to the placebo group.

In both SAD and MAD parts, the SSS, MOAA/S, and C-SSRS scales were also assessed as safety parameters. No clinically significant safety risks related to sedation, somnolence, or suicidal ideation were indicated by comparisons of post-dose scores with baseline, together with the investigator’s clinical evaluation.

### Efficacy

**Fig. 2:**
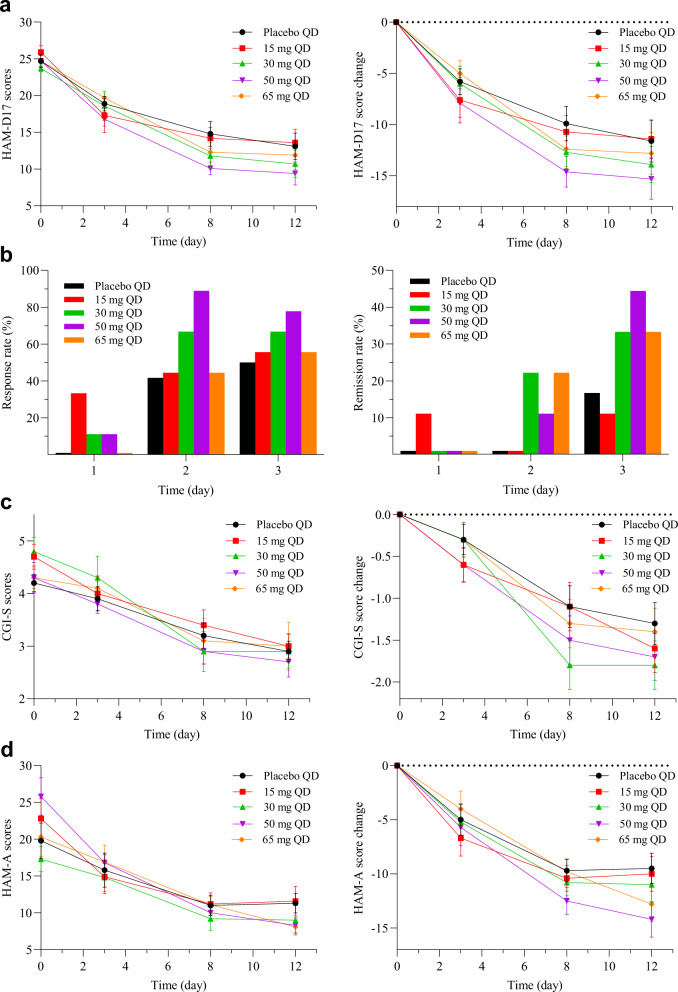
Efficacy endpoints. **a** 17-item Hamilton Depression Rating Scale (HAM-D17) scores (left panel) and change from baseline (right panel); **b** HAM-D17 response rate (left panel) and remission rate (right panel); **c** Clinical Global Impression-Severity (CGI-S) scores (left panel) and change from baseline (right panel); **d** Hamilton Rating Scale for Anxiety (HAM-A) scores (left panel) and change from baseline (right panel)

The principal efficacy outcomes are detailed in Additional File 1: Table S2. In brief, the mean HAM-D17 scores at baseline were 24.7, 25.9, 23.7, 24.7, and 24.7 for the placebo, 15 mg, 30 mg, 50 mg, and 65 mg dose cohorts, respectively. After seven consecutive daily doses of HS-10353, the least-squares mean differences (95% CI) in HAM-D17 score change from baseline versus placebo were −0.8 (−5.1, 3.4), −2.9 (−7.1, 1.4), −4.7 (−8.9, −0.5) and −2.5 (−6.7, 1.7) for HS-10353 15 mg, 30 mg, 50 mg, and 65 mg cohorts, respectively, on day 8 (Fig. 2a). HS-10353 demonstrated a dose- dependent reduction in HAM-D17 scores for doses ranging from 15 mg to 50 mg, with the most significant reduction observed in the 50 mg dose cohort. The differences in HAM-D17 response rates versus placebo at day 8 were 2.8%, 25.0%, 47.2% and 2.8%, for HS-10353 15 mg, 30 mg, 50 mg, and 65 mg dose cohorts, respectively (Fig. 2b). Notably, the HAM-D17 response at day 8 was lower in the 65 mg cohort compared to the 50 mg cohort. HAM-D17 remission rates were 0 in both HS-10353 15 mg and placebo cohorts at day 8, and the differences in remission rates versus placebo were 22.2%, 11.1% and 22.2% for HS-10353 30 mg, 50 mg, and 65 mg dose cohorts, respectively (Fig. 2b). The mean CGI-S scores at baseline were 4.2, 4.7, 4.8, 4.3, and 4.3 for the placebo, 15 mg, 30 mg, 50 mg, and 65 mg dose groups, respectively. Following seven consecutive daily doses of HS-10353, the least-squares mean differences (95% CI) in CGI-S change from baseline versus placebo at day 8 were 0.0 (−0.8, 0.7), −0.7 (−1.5, 0.1), −0.4 (−1.2, 0.4), and −0.2 (−0.9, 0.6) for HS-10353 15 mg, 30 mg, 50 mg, and 65 mg dose groups, respectively (Fig. 2c). The mean HAM-A scores at baseline were 19.8, 22.8, 17.3, 25.8, and 20.3 for the placebo, 15 mg, 30 mg, 50 mg, and 65 mg dose groups, respectively. After seven consecutive daily doses of HS-10353, the least-squares mean differences (95% CI) in HAM-A changes from baseline versus placebo at day 8 were −0.7 (−3.9,2.5), −1.1 (−4.3,2.1), −2.8 (−6.1,0.5), and −0.1 (−3.2,3.1) for HS-10353 15 mg, 30 mg, 50 mg, and 65 mg dose groups, respectively (Fig. 2d).

### Pharmacokinetics

**Fig. 3:**
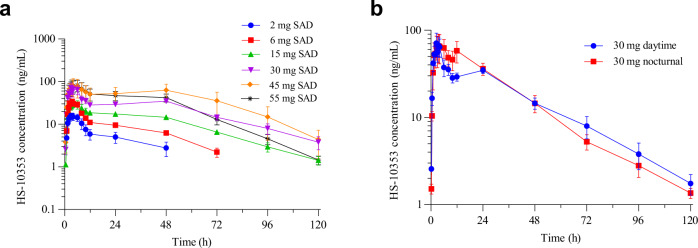
Plasma concertation profiles of HS-10353 in SAD part (semilogarithmic scale). **a** plasma concentration–time curves following single dose administration. **b** chronopharmacology plasma concentration–time curves following single dose administration

As illustrated in Fig. 3, following a single oral fasting administration, HS-10353 was rapidly absorbed, with peak plasma concentrations observed between 1.75- and 3.75-hours. Subsequently, HS-10353 concentrations displayed a biphasic PK profile, characterized by a relatively rapid distribution phase followed by a slower elimination phase. As summarized in Table 5, the mean t_1/2_ ranged from approximately 14.45 to 21.80 h across all doses. Within the fasting dose range of 2 to 45 mg, plasma concentrations and exposure parameters (C_max_, AUC_0-t_, and AUC_0-∞_) increased in a dose-dependent manner. Further increasing dose from 45 mg to 55 mg, no increases in fasting expsoure (C_max_, AUC_0-t_, and AUC_0-∞_) was observed. The power model-derived estimated regression coefficients and 90% confidence intervals (90% CI) for the dose proportionality constant *β* of HS-10353 were as follows: 0.4573 (90% CI: 0.3010–0.6136) for C_max_, 0.8557 (90%CI: 0.7191–0.9922) for AUC_0-t_, and 0.8309 (90% CI: 0.6953–0.9664) for AUC_0-∞_ (Additional File 1: Fig. S1), within the fasting dose range of 2-45 mg, suggesting a possible saturation of absorption at high doses. Additionally, no significant differences in PK parameters were observed between nighttime and daytime cohorts following a single oral 30 mg dose of HS-10353.

**Table 5:** Pharmacokinetic parameters of HS-10353 in the SAD part (pharmacokinetic parameter set)

Parameters		2 mg (*N* = 6)	6 mg (*N* = 6)	15 mg (*N* = 6)	30 mg daytime (*N* = 6)	30 mg nighttime (*N* = 6)	45 mg (*N* = 6)	55 mg (*N* = 6)
C_max_ (ng/mL)	Mean (SD)	16.50 (6.963)	35.83 (12.50)	32.52 (15.89)	77.27 (50.69)	91.65 (53.67)	91.33 (87.25)	90.32 (27.09)
T_max_ (h)	Median (Min, Max)	1.750 (1.500–4.000)	3.000 (1.500–3.000)	3.500 (2.000–12.00)	3.000 (2.500–24.00)	3.750 (2.000–12.00)	3.000 (2.000–24.00)	3.750 (3.000–6.000)
AUC_0-t_ (h·ng/mL)	Mean (SD)	196.6 (157.4)	409.8 (78.74)	812.5 (172.2)	1859 (250.5)	2082 (900.3)	3317 (3836)	2092 (446.5)
AUC_0-∞_ (h·ng/mL)^*^	Mean (SD)	227.8 (161.0)	471.2 (85.66)	926.1 (165.2)	1935 (261.8)	2124 (907.0)	3854 (4306)	2137 (449.2)
t_1/2_ (h)^*^	Mean (SD)	14.57 (9.123)	17.28 (8.430)	21.80 (6.902)	20.14 (5.872)	19.43 (3.583)	14.45 (2.519)	16.20 (1.608)
CL/F (L/h)*	Mean (SD)	11.37 (4.784)	13.10 (2.432)	16.61 (2.886)	15.74 (2.066)	16.12 (6.052)	24.41 (18.46)	27.10 (7.853)
V_d_/F (L)^*^	Mean (SD)	209.2 (111.9)	307.7 (95.78)	509.4 (93.20)	450.8 (122.7)	466.3 (242.4)	483.2 (345.0)	641.5 (242.0)
MRT (h)^*^	Mean (SD)	19.23 (12.02)	23.90 (13.25)	33.23 (8.662)	35.63 (10.81)	30.15 (5.204)	35.19 (14.33)	25.69 (1.694)

^*^Two participants (in 15 mg and 45 mg groups) were excluded from the summary statistics and statistical analyses related to this PK parameter because an elimination rate constant could not be accurately estimated

C_max_, the maximum observed plasma concentration; T_max_, the time to reach C_max_; AUC_0-t_, the area under the plasma concentration–time curves from 0 to t h; AUC_0-∞_, the area under the plasma concentration-time curves from 0 h to infinity; t_1/2_, terminal time of half-life; CL/F, apparent elimination clearance over bioavailability; V_d_/F, apparent volume of distribution; MRT, mean residence time

**Fig. 4:**
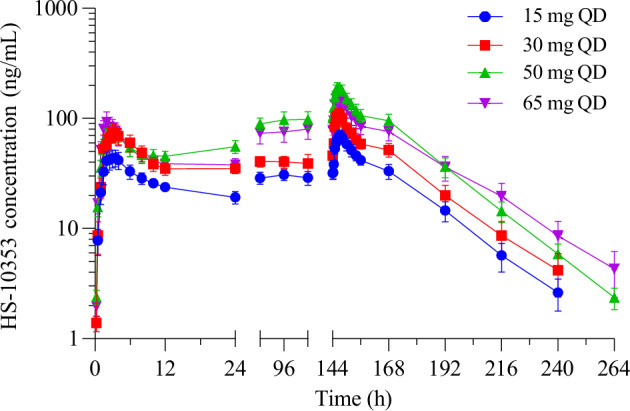
Plasma concertation profiles of HS-10353 in MAD part (semilogarithmic scale)

In the MAD part, steady-state fasting plasma concentrations of HS-10353 were achieved after 5 days of dosing (Fig. 4). Daily fasting administration for up to 7 days resulted in an average accumulation ratio of 1.733–2.573 for AUC_ss, 0-τ_ and 1.316–2.650 for C_ss, max_, respectively (Table 6). Following 7 consecutive daily doses, the mean t_1/2_ ranged from 17.11 to 19.26 h across all dose groups. Exposure parameters, including C_ss, max_, C_ss, min_, C_ss, av_, and AUC_ss, 0-τ_ increased with increasing doses of HS-10353 over the dose range of 15-50 mg. However, at the 65 mg dose level, exposure was lower compared to 50 mg, suggesting a saturation of absorption. Power model analysis of dose proportionality indicated that the *β* values and their respective 90% CIs for C_ss, max_ and AUC_ss, 0-τ_ were 0.8316 (90% CI: 0.5533–1.1098) and 0.7997 (90% CI: 0.5519–1.0475), respectively, within the 15-50 mg dose range (Additional File 1: Fig. S2).

**Table 6:** Pharmacokinetic parameters of HS-10353 in the MAD part (pharmacokinetic parameter set)

Characteristics		15 mg QD (*N* = 9)	30 mg QD (*N* = 9)	50 mg QD (*N* = 9)	65 mg QD (*N* = 9)
C_ss,max_ (ng/mL)	Mean (SD)	74.44 (28.18)	112.6 (50.95)	197.8 (61.00)	160.7 (76.14)
C_ss,min_ (ng/mL)	Mean (SD)	29.43 (12.16)	43.49 (23.24)	90.94 (41.99)	69.31 (37.22)
C_ss,av_ (ng/mL)	Mean (SD)	45.87 (15.16)	67.44 (24.86)	123.9 (46.63)	99.08 (45.42)
T_ss,max_ (h)	Median (Min, Max)	3.000 (2.000–6.000)	3.000 (1.000–3.500)	3.000 (1.500–6.000)	2.500 (1.500–4.000)
AUC_ss_ (h·ng/mL)	Mean (SD)	1101 (363.8)	1619 (596.7)	2973 (1119)	2378 (1090)
t_1/2_ (h)	Mean (SD)	17.11 (4.950)	18.03 (7.572)	17.83 (2.330)	19.26 (7.993)
CL_ss_/F (L/h)	Mean (SD)	15.38 (6.399)	20.40 (5.890)	19.44 (8.448)	36.26 (24.49)
DF (%)	Mean (SD)	98.32 (52.54)	100.8 (36.46)	93.25 (31.42)	98.42 (46.02)
R_AUC_	Mean (SD)	1.838 (0.3015)	1.733 (0.3966)	2.573 (1.834)	2.059 (0.5783)
R_Cmax_	Mean (SD)	1.629 (0.5130)	1.316 (0.3007)	2.650 (1.260)	1.743 (0.5929)

*C*_*ss, max*_, steady-state maximum concentration, *C*_*ss, min*_, steady-state minimum concentration, *C*_*ss, av*_, steady-state average concentration, *T*_*ss, max*_, time to maximum concentration at steady state, *AUC*_*ss, 0-τ*_, area under the steady-state plasma concentration-time curve, *R*_*ac*_, accumulation ratio of multiple doses, *DF,* degree of fluctuation

## Discussion

Despite the widespread availability of antidepressant therapies, approximately one-third of patients exhibit inadequate responses to current treatments [1, 18, 24]. Consequently, there is an urgent need to develop novel antidepressant strategies. GABA, a naturally occurring neurotransmitter, plays a crucial role in the central nervous system. High expectations have been placed on GABA_A_ receptor positive allosteric modulators for improving treatment outcomes in PPD patients. In addition to zuranolone, which is currently available on the market, several other GABA_A_ receptor modulators are under development globally, including HS-10353.

The present study suggested that HS-10353 was generally safe in healthy volunteers following single doses ranging from 2 to 55 mg, as well as in MDD patients following multiple doses in the range of 15 to 65 mg. Unlike currently marketed antidepressants (e.g., MAOIs, SSRIs and SNRIs) associated with chronic tolerability concerns, HS-10353, which acts as a positive allosteric modulator of GABA_A_ receptor, demonstrated favorable short-term safety in this phase 1 study.

Mechanistically, HS-10353 may cause sedation and somnolence. In this study, sedative and somnolence were evaluated by MOAA/S scale and SSS scale combined with the clinical evaluation. No adverse reactions of sedation or somnolence were observed in the SAD part. The incidence of somnolence with HS-10353 versus placebo in the MAD part was 8.3% vs 16.7%, and no side effects of sedation were noted. In the SAD part, within the HS-10353 group, ALT levels increased in 3 participants (8.3%), AST levels increased in 2 participants (5.6%), γ-glutamyltransferase levels increased in 1 participant (2.8%), alkaline phosphatase levels increased in 1 participant (2.8%), and fibrinogen levels increased in 1 participant (2.8%). Two cases of pruritus were reported in 6 mg and 15 mg cohort of SAD part, one is accompanied by slight increase in ALT and AST, but none was observed with elevation of bilirubin. All of these adverse events were classified as mild, and none required medication to return to normal levels. In the MAD part, 2 patients (5.6%) in the HS-10353 group experienced elevations in ALT, while 1 patient (2.8%) was reported to have mild “hepatic failure” by investigator. This patient had slight increase in ALT (63 U/L) and AST (47 U/L) five days after last dosing, without elevation of bilirubin, GGT, alkaline phosphatase or lactate dehydrogenase, and ALT and AST returned to normal within 9 days. The elevations of ALT, AST and GGT in both SAD and MAD studies were all within twice of upper normal limit. The magnitude of elevation in HS-10353 cohorts was comparable to that in placebo cohort. These elevations occurred across day 3 to day 12 after study treatment. No elevation of bilirubin was accompanied.

In the SAD part, fasting C_max_ and AUC values increased approximately dose proportionally across the 2-45 mg dose range. However, the exposure in the 55 mg dose group was observed to be lower than that in the 45 mg dose group. Similarly, fasting exposures in the MAD cohorts for 15 mg to 50 mg doses appeared to increase dose-proportionally, but the exposure in the 65 mg dose group was found to be lower than that in the 50 mg dose group. This indicates that HS-10353 is likely to reach absorption saturation at fasting dose levels > 45 mg, the fraction absorbed no longer increases with dose and leading to a decrease in bioavailability (F), which results in reduced exposure. Physicochemical profiles show that HS-10353 has a low solubility in water and is categorized as a BCS Class II compound. A comprehensive assessment suggests that the potential saturation absorption of HS-10353 at higher dosing level may be due to the low solubility of the compound. In the MAD part, PK results showed rapid drug absorption with a median T_max_ ranging from 2.5 to 3.0 h. The terminal half-life ranged from approximately 17 to 19 h. Minimal accumulation was noted after repeat dosing, suggesting that patients would reach target concentrations relatively quickly after initiation of treatment.

There was a dose-response relationship for HS-10353 in MDD patients. A placebo effect was observed during the MAD part, characterized by reductions in HAM-D17 scores from baseline in the placebo group at Day 3, Day 8, and Day 12 of −5.8, −9.9, and −11.6, respectively. The magnitude of the placebo effect increased with the number of doses administered and persisted through Day 12.

HS-10353 showed a reduction in HAM-D17 scores compared to placebo as early as day 3 in most dose groups. This finding was consistent with the results of the PK analysis, where exposure was lower in the higher dose group possibly due to an absorption saturation, indicating a potential exposure-efficacy relationship of HS-10353. The improvements over placebo on HAM-D17 scale score from baseline, response rate and remission rate were observed with a dose dependence manner. The absolute reduction from baseline in the HAM-D17 scale score was most pronounced on day 8 for patients receiving doses of 15 to 65 mg. Notably, the 50 mg treatment group showed a difference of −4.7 (95% CI: −8.9 to −0.5) in change from baseline of HAM-D17 score compared to placebo. While the therapeutic effects of current first-line antidepressants typically manifest over a period of several weeks, HS-10353 showed a reduction in HAM-D17 scores within one week in this study. These early efficacy signals support further investigation of HS-10353 in additional studies, including a phase 2 study in PPD (NCT05937867).

Despite the encouraging results, our study has limitations. The small sample size and very short treatment duration in this Phase 1 trial limits the power to balance demographic baselines and precludes definitive efficacy conclusion. Subsequently, the sample size will be expanded and the optimal treatment duration will be warranted in the Phase 2 study to explore the antidepressant efficacy and safety at different dose levels.

## Conclusions

Single and multiple oral doses of HS-10353 were safe and well tolerated, with favorable fasted PK properties suitable for once-daily administration. HS-10353 showed a reduction in HAM-D17 scores compared to placebo within one week in MDD patients. These findings collectively support continued development of HS-10353.

## Supplementary information

Additional File1-1 Table S1. Abnormal values for ALT, AST, GGT, ALP and fibrinogen in SAD study. Table S2. Efficacy endpoints assessed at Day 8 after administration of HS-10353 once daily for 1 week (full analysis set). Fig. S1 Dose proportionality and linearity in SAD part (AUC_0-∞_ over HS-10353 dose). Fig. S2 Dose proportionality and linearity in MAD part (AUC_SS_ over HS-10353 dose)

## Data Availability

The datasets used and analyzed in this study are available from the corresponding author upon reasonable request.

## Abbreviations

AE: adverse event
AUC: area under the plasma concentration-time curve
BMI: Body Mass Index
CGI-S: Clinical Global Impression-Severity
CL/F: apparent clearance
C_max_: maximum observed plasma concentration
CNS: central nervous system
C-SSRS: Columbia-Suicide Severity Rating Scale
DSM-5: Diagnostic and Statistical Manual of Mental Disorders, Fifth Edition
ECG: electrocardiogram
FDA: US Food and Drug Administration
GABA: γ-aminobutyric acid
HAM-A: Hamilton Rating Scale for Anxiety
HAM-D17: Hamilton Rating Scale for Depression
MAD: multiple ascending dose
MDD: major depressive disorder
M.I.N.I. 7.0.2: Mini International Neuropsychiatric Interview
MOAA/S: Modified Observer’s Assessment of Alertness/Sedation
MRT: mean residence time
NMPA: China’s National Medical Products Administration
PK: pharmacokinetics
PPD: postpartum depression
QTcF: QTc interval corrected by Fridericia’s formula
R_ac_: accumulation ratio
SAD: single ascending dose
SAE: serious adverse event
SNRI: serotonin–norepinephrine reuptake inhibitor
SSRI: selective serotonin reuptake inhibitor
SSS: Stanford Sleepiness Scale
t_1/2_: terminal phase elimination half-life
T_max_: time to reach C_max_
TEAE: treatment emergent adverse event
TRD: treatment-resistant depression
V_d_/F: apparent volume of distribution
WHO: World Health Organization.
