# Supplementary material for: Phase 1 randomized trial of HS-10353, a novel GABA(A) positive allosteric modulator for treatment of major depressive disorder
Source: BMC Med. 2026 May 21;24:399. doi: 10.1186/s12916-026-04926-5 (PMC13374185; doi:10.1186/s12916-026-04926-5)
Supplement: Supplementary file 1 — Additional File1-1 Table S1. Abnormal values for ALT, AST, GGT, ALP and fibrinogen in SAD study. Table S2. Efficacy endpoints assessed at Day 8 after administration of HS-10353 once daily for 1 week (full analysis set). Fig. S1 Dose proportionality and linearity in SAD part (AUC0-∞ over HS-10353 dose). Fig. S2 Dose proportionality and linearity in MAD part (AUCSS over HS-10353 dose) [file 12916_2026_4926_MOESM1_ESM.docx]

**Table S1. Abnormal values for ALT, AST, GGT, ALP and fibrinogen in SAD study**

| **Participant** | **Group** | **Item (Unit)** | **Visit** | **Results** | **Clinical Assessment** | **Reference Range** |
| --- | --- | --- | --- | --- | --- | --- |
| P-001 | SAD 6 mg | ALT (U/L) | Baseline | 55 | NCS | 0-50 |
| P-002 | SAD 6 mg | ALT (U/L) | Baseline | 42 | NCS | 0-40 |
| P-003 | SAD 15 mg | ALT (U/L) | Day6 | 85 | CS | 0-50 |
| P-004 | SAD Placebo | ALT (U/L) | Day3 | 73 | CS | 0-50 |
| P-004 | SAD Placebo | ALT (U/L) | Day6 | 57 | NCS | 0-50 |
| P-005 | SAD 45 mg | ALT (U/L) | Day6 | 79 | CS | 0-40 |
| P-006 | SAD 55 mg | ALT (U/L) | Day3 | 54 | NCS | 0-50 |
| P-006 | SAD 55 mg | ALT (U/L) | Day6 | 93 | CS | 0-50 |
| P-003 | SAD 15 mg | AST (U/L) | Day6 | 59 | CS | 0-40 |
| P-005 | SAD 45 mg | AST (U/L) | Day6 | 45 | CS | 0-35 |
| P-006 | SAD 55 mg | AST (U/L) | Day3 | 44 | NCS | 0-40 |
| P-006 | SAD 55 mg | AST (U/L) | Day6 | 43 | NCS | 0-40 |
| P-007 | SAD 6 mg | GGT (U/L) | Baseline | 76 | NCS | 0-60 |
| P-007 | SAD 6 mg | GGT (U/L) | Day3 | 70 | NCS | 0-60 |
| P-007 | SAD 6 mg | GGT (U/L) | Day6 | 82 | NCS | 0-60 |
| P-001 | SAD 6 mg | GGT (U/L) | Baseline | 94 | NCS | 0-60 |
| P-001 | SAD 6 mg | GGT (U/L) | Day3 | 60 | NCS | 0-60 |
| P-005 | SAD 45 mg | GGT (U/L) | Day6 | 74 | CS | 0-45 |
| P-005 | SAD 45 mg | GGT (U/L) | 8±1 days after discharge | 47 | NCS | 0-45 |
| P-006 | SAD 55 mg | GGT (U/L) | Day6 | 61 | NCS | 0-60 |
| P-005 | SAD 45 mg | ALP (U/L) | Day3 | 101 | NCS | 35-100 |
| P-005 | SAD 45 mg | ALP (U/L) | Day6 | 143 | CS | 35-100 |
| P-005 | SAD 45 mg | ALP (U/L) | 8±1 days after discharge | 108 | NCS | 35-100 |
| P-005 | SAD 45 mg | Fibrinogen (g/L) | Unscheduled visit | 8.16 | CS | 2-4 |
| P-005 | SAD 45 mg | Fibrinogen (g/L) | Unscheduled visit | 8.52 | CS | 2-4 |
| P-005 | SAD 45 mg | Fibrinogen (g/L) | Unscheduled visit | 6.45 | CS | 2-4 |
| Participant identifiers were replaced with anonymized codes (e.g., P-001, P-002).  CS, clinical significance; NCS, non-clinical significance. | | | | | | |

**Table S2. Efficacy endpoints assessed at Day 8 after administration of HS-10353 once daily for 1 week (full analysis set)**

| **Efficacy endpoints** | **15 mg QD**  **(N = 9)** | **30 mg QD**  **(N = 9)** | **50 mg QD**  **(N = 9)** | **65 mg QD**  **(N = 9)** | **Placebo QD**  **(N = 12)** |
| --- | --- | --- | --- | --- | --- |
| **Change from baseline in HAM-D17 score at D8** | | | | | |
| LSM (95% CI) | -10.7 (-13.9, -7.4) | -12.7(-16.0, -9.5) | -14.6(-17.8, -11.4) | -12.4(-15.6, -9.2) | -9.9(-12.6, -7.1) |
| LSMD (95% CI) | -0.8 (-5.1, 3.4) | -2.9(-7.1, 1.4) | -4.7 (-8.9, -0.5) | -2.5 (-6.7, 1.7) |  |
| **Reduction of >50% from baseline in HAM-D17 score at D8** | | | | | |
| % (95% CI) | 44.4 (13.7,78.8) | 66.7 (29.9,92.5) | 88.9 (51.8,99.7) | 44.4 (13.7,78.8) | 41.7 (15.2,72.3) |
| Difference | 2.8 (-40.0,45.6) | 25.0 (-16.6,66.6) | 47.2 (12.6,81.9) | 2.8 (-40.0,45.6) |  |
| **HAM-D17 score ≤7 at D8** | | | | | |
| % (95% CI) | 0 (0.0,33.6) | 22.2 (2.8,60.0) | 11.1 (0.3,48.2) | 22.2 (2.8,60.0) | 0 (0.0,26.5) |
| Difference | NA (NA,NA) | 22.2 (-4.9,49.4) | 11.1 (-9.4,31.6) | 22.2 (-4.9,49.4) |  |
| **Change from baseline in HAM-A score at D8** | | | | | |
| LSM (95% CI) | -10.4 (-12.8, -8.0) | -10.8 (-13.2, -8.3) | -12.5 (-15.0, -10.0) | -9.8 (-12.2, -7.4) | -9.7 (-11.8, -7.6) |
| LSMD (95% CI) | -0.7 (-3.9,2.5) | -1.1 (-4.3,2.1) | -2.8 (-6.1,0.5) | -0.1 (-3.2,3.1) |  |
| **Change from baseline in CGI-S score at D8** | | | | | |
| LSM (95% CI) | -1.1 (-1.7, -0.5) | -1.8 (-2.4, -1.2) | -1.5 (-2.1, -0.9) | -1.3 (-1.8, -0.7) | -1.1 (-1.6, -0.6) |
| LSMD (95% CI) | 0.0 (-0.8, 0.7) | -0.7 (-1.5, 0.1) | -0.4 (-1.2, 0.4) | -0.2 (-0.9, 0.6) |  |

All differences were compared versus the placebo cohort. The least-squares mean changes from baseline in the scores on the HAM-D17, HAM-A, and CGI-S, which were calculated with the use of a mixed-effects model for repeated measures. The 95% CIs of response and remission rates were estimated using the Clopper-Pearson method and the 95% CIs of risk differences were calculated based on normal approximation. CGI-S, Clinical Global Impression-Severity score; CI, confidence interval; HAM-A, Hamilton Anxiety Scale score; HAM-D17, Hamilton Depression Rating Scale score; LSM, least squares mean; LSMD, least squares mean difference.

**Fig. S1 Dose proportionality and linearity in SAD part (AUC_0-∞_ over HS-10353 dose).**

**Fig. S2 Dose proportionality and linearity in MAD part (AUC_SS_ over HS-10353 dose).**
